# Colonial and post-colonial collections in Portugal: reconstructing trajectories and rethinking narratives

**DOI:** 10.1590/S0104-59702025000100032en

**Published:** 2025-09-08

**Authors:** Elisabete Pereira, Catarina Simões, Quintino Lopes

**Affiliations:** i IN2PAST/Instituto de História Contemporânea/Universidade de Évora. Évora – Portugal; ii CHAM - Centro de Humanidades, Faculdade de Ciências Sociais e Humanas/Universidade Nova de Lisboa. Lisboa – Portugal; iii IN2PAST/Instituto de História Contemporânea/Universidade de Évora. Évora – Portugal

On the eve of the fiftieth anniversary of April 25, 1974, the date of the revolution that established democracy in Portugal and began the process of decolonizing Portuguese territories in Africa and Asia, Portuguese President Marcelo Rebelo de Sousa declared the need for reparations for colonialism. He mentioned “goods that were plundered and not returned, when it has been proven that they were plundered” (DN/Lusa, 24 abr. 2024). A few days later, when journalists continued to push the subject, Rebelo de Sousa added: “We can’t sweep this under the carpet or put it in a drawer. We have an obligation to pilot, to lead this process [of reparations]” (DN/Lusa, 27 abr. 2024). He also mentioned the need to continue “the process of surveying the assets that are the heritage of the former colonies in Portugal, [which was] started by the previous government, in order to return them later” (RFI com Lusa, 29 abr. 2024).

The previous year, in 2023, at a session welcoming Brazilian President Lula da Silva (which also preceded the ceremony commemorating the anniversary of April 25 in the National Assembly), the Portuguese president had argued that Portugal owed an apology and should assume full responsibility for the exploitation and slavery that occurred during the colonial period (SIC Notícias, 26 abr. 2023).

On all these occasions, the president’s statements had a considerable impact on the media, abroad but especially in Portugal, where heated debates were held with academics, museologists, journalists and public figures for several days. They discussed Rebelo de Souza’s position and statements, the possible “reparations,” and what would be necessary to carry them out. After a few weeks, the issue was gradually forgotten.

The survey (even if only partial) of the former colonies’ assets in Portugal mentioned by the president has not been made public. What are the plundered objects Rebelo de Sousa referred to? How many are there? Where are they? Who identified them, and what historical sources show that they were plundered or acquired as a result of pressure and the power imbalance typical of the colonial period? As Clémentine Deliss (2020) points out, we face the incalculability of restitution, even more so when it is not clear what we are dealing with.

The lack of knowledge about and interest in these assets in Portugal is evident in the “Inquérito à presença de património proveniente de territórios não europeus nos museus portugueses” [Survey on the presence of heritage from non-European territories in Portuguese museums] conducted by the Portuguese National Commission of the International Council of Museums in 2021. Of the more than four hundred museum institutions in Portugal, 67 responded to the survey. From their answers, we know that some have non-European collections and that many of these have documentation associated with the objects in question, which means that data exists which can lead to a deeper understanding of their provenance and the contexts in which they were acquired. The survey also revealed a “lack of a detailed inventory” as well as “insufficient study of these collections” in a significant portion of the museums that took part in the study (Amaro, Felismino, 2021, p.133).

As Clémentine Deliss (former director of the Weltkulturen Museum in Frankfurt from 2010 to 2015) has stated, the museums that promoted the establishment of these collections and exhibitions of artifacts from cultural contexts under colonial rule sought to shape visitors’ visions of Western and European progress (Deliss, 2020, p.15-16). The exhibitions that once celebrated “Western civilizational progress” can now serve as proof of the violence and injustice experienced by those who were on the other side of this progress (Sterling, 2024, p.3). To do so, it is necessary to understand the epistemologies that produced these collections and are still reflected today in the way collections are organized, and often in the way they are studied and presented to the public. Decoding the thousands of artifacts in the museums, including access to information about colonial practices and the contexts within which they were collected, will require contacting the descendants of communities and their diasporas, as well as accessing the many areas where these institutions store their objects. Often kept in inaccessible storage units, these artifacts that were acquired, stolen, looted or ransomed in the name of science, trade or diplomatic exchanges were inscribed in the historical annals of the institutions and remain there, invisible and silenced (Deliss, 2020, p.16). From these records, historical information can be retrieved that is fundamental for conducting provenance studies and reconstructing the trajectories of cultural artifacts that can contribute to new historical narratives, museological discourses, restitutions or reparations.

In Europe, much has been published in recent years about the colonial origins and legacies of old ethnological and imperial museums. Attempts to decolonize by opening museums up to research through collaborative projects with universities and communities and promoting revision of content and Eurocentric and racialized language replicated by museum institutions over the decades are just a few examples of projects that have been undertaken. At the same time, new lines of research and centers are springing up on the African continent, particularly in Dakar, Cape Town and Nairobi, where artists and curators are successfully redefining museology and its practices. Various African curators, artists and activists have been working in European institutions with the challenge of rethinking “ethnographic collections,” including those from their countries of origin. Specialized curatorial positions are being created, as in the case of the University of Manchester Museum, where there is a “curator of living cultures”.^1^

In several European countries, debates on provenance research have sparked a panoply of scientific and museological initiatives, along with the formulation of guidelines and even legislation to coordinate management, research and restitution of colonial collections. This resurgence of the debate on collections with colonial origins in museums in the Global North is rooted in a generational shift and distancing from the colonial period (Van Beurden, Gondola, Lacaille, 2023, p.23). Of course, there is a long history of requests for restitution that have consistently called attention to the problematic origin of colonial collections. This question has also connected with growing academic interest in the ways colonial pasts survive today, and how we can confront the colonial legacy in the museum through various initiatives, namely exhibitions like the one that opened in 2025 at the Santos Rocha Municipal Museum in Portugal.^2^ The reinvention and reestablishment of European ethnographic museums has also influenced this resurgence. The creation of the Humboldt Forum in Berlin and the Quai Branly Museum in Paris and the renovation of the Africa Museum in Tervuren sparked wide criticism of how their exhibitions maintained colonial paradigms of knowledge. In Portugal, in 2024 the National Museum of Ethnology inaugurated the exhibition “Deconstruir o colonialismo, descolonizar o imaginário” [Deconstructing colonialism, decolonizing the imaginary] to contribute to a renovation of knowledge about Portugal’s colonial history. However, the institution is still working to present its collections with a renewed vision.

The articles collected in this supplemental issue of *História, Ciências, Saúde - Manguinhos* entitled “Coleções coloniais e pós-coloniais em Portugal: reconstruir trajetórias e repensar narrativas” [Colonial and post-colonial collections in Portugal: reconstituting trajectories and rethinking narratives] show the growing attention that has been paid in recent years to the need to revisit, question and reinterpret the colonial legacies present in Portugal’s museums and scientific institutions. The texts reflect work being carried out on the legacy of various Portuguese institutions (such as the National Museum of Natural History and Science, the Santos Rocha Municipal Museum, the National Museum of Archaeology and the University of Porto’s Museum of Natural History and Science), showing how in many cases their collections were shaped by the dynamics of occupation, proselytization, trade and colonial domination. We also wish to highlight studies currently underway on collections obtained in territories ruled by the Portuguese through missionary activities, and how research on the way these collections arrived at these institutions and the power networks associated with their creation allows us to uncover their origins, their contexts of acquisition, and the permanence of Eurocentric discourses. The articles also highlight growing attention paid to contemporary African art and to artists of African descent whose works reshape the ways in which colonial history and memory are represented. The component of private collecting is also included in this supplement as a way of understanding the motivations, contexts and impacts of these collections, especially when they belong to artists, and how they dialog with Portuguese colonial and post-colonial histories.

These texts are the result of inter-institutional collaboration and dedication on the part of researchers who are committed to the need to revise dominant histories, and are intended to appeal to the social responsibility of museums and scientific institutions with regard to their colonial heritage.

## NOTES

1 Anthropologist Njabulo Chipanguro, originally from Zimbabwe, was a co-curator of colonial collections from 2022 to 2025 at the Manchester Museum. He holds a doctorate in Anthropology from the University of the Witwatersrand in Johannesburg, South Africa, and previously worked as a curator in the archaeology department of the National Museums and Monuments of Zimbabwe for ten years. See Chipangura, Mataga (2021).

2 “Facing the Colonial Legacy in the Museum” was an exhibition organized by a project entitled “TRANSMAT - Transnational Materialities (1850-1930): Reconstituting Collections and Connecting Histories” (PTDC/FER-HFC/2793/2020) which took place in March 2025 at the Santos Rocha Municipal Museum in Figueira da Foz, Portugal. For four years the TRANSMAT Project investigated the transnational collections of the Museu Municipal Santos Rocha and Museu Nacional de Arqueologia (for more on the project, see Pereira, Carvalho, Cardoso, 2021). The research was conducted by the Instituto de História Contemporânea, which receives national funds from the Foundation for Science and Technology (Fundação para a Ciência e a Tecnologia, FCT), I.P., within the scope of projects UID/04209 and LA/P/0132/2020 (DOI 10.54499/LA/P/0132/2020). Elisabete Pereira receives funding from CEEC FCT (10.54499/2022.04155.CEECIND/CP1734/CT0004), and Quintino Lopes receives funding from CEEC-FCT (10.54499/2021.00906.CEECIND/CP1670/CT0005).
