# Supplementary material for: Colonial and post-colonial collections in Portugal: reconstructing trajectories and rethinking narratives
Source: Hist Cienc Saude Manguinhos. 2025 Sep 8;32(Suppl 1):e2025032. doi: 10.1590/S0104-59702025000100032en (PMC12424435; doi:10.1590/S0104-59702025000100032en)
Supplement: Translation

# Translation: Coleções coloniais e pós-coloniais em Portugal: reconstituir trajetórias e repensar narrativas

**DOI:** 10.1590/S0104-59702025000100032

**Authors:** Elisabete Pereira, Catarina Simões, Quintino Lopes

**Affiliations:** i IN2PAST/Instituto de História Contemporânea/Universidade de Évora. Évora – Portugal; ii CHAM – Centro de Humanidades, Faculdade de Ciências Sociais e Humanas/Universidade Nova de Lisboa. Lisboa – Portugal; iii IN2PAST/Instituto de História Contemporânea/Universidade de Évora. Évora – Portugal

Na véspera da comemoração dos 50 anos do 25 de Abril de 1974, a revolução que implantou a democracia em Portugal e que iniciou o processo de descolonização de territórios portugueses em África e na Ásia, o presidente da República portuguesa, Marcelo Rebelo de Sousa, declarou a necessidade de reparações relativas ao colonialismo. Mencionou “os bens espoliados e que não foram devolvidos, quando se provou que eram espoliados” (DN/Lusa, 24 abr. 2024). Aos jornalistas que o interpelavam continuamente sobre o assunto, Marcelo Rebelo de Sousa acrescentou dias depois: “Não podemos meter isto debaixo do tapete ou dentro da gaveta. Temos obrigação de pilotar, de liderar este processo” (DN/Lusa, 27 abr. 2024). O presidente mencionou também a necessidade de continuação “do processo de levantamento dos bens patrimoniais das ex-colónias em Portugal, iniciado pelo anterior governo, para posteriormente devolvê-los” (RFI com Lusa, 29 abr. 2024).

No ano anterior, em 2023, numa sessão de boas-vindas ao presidente da República Federativa do Brasil, Lula da Silva, que antecedeu também a sessão solene comemorativa do aniversário do 25 de Abril na Assembleia da República, o presidente português defendera que Portugal devia um pedido de desculpa e que deveria assumir plenamente a responsabilidade pela exploração e pela escravatura no período colonial (SIC Notícias, 26 abr. 2023).

Em todas essas ocasiões as suas declarações assumiram um impacto considerável nos meios de comunicação social, nomeadamente estrangeiros, mas especialmente em Portugal, onde durante alguns dias se promoveram acesos debates com académicos, museólogos, jornalistas e figuras públicas. Discutiram-se a posição e as declarações do presidente, as possíveis “reparações”, as condições necessárias para promovê-las. Passadas poucas semanas, a questão foi progressivamente esquecida.

O levantamento (mesmo que parcial) dos bens patrimoniais das ex-colónias em Portugal, mencionado pelo presidente da República, não é público. Quais são os objetos espoliados mencionados pelo presidente da República? Quantos são? Onde se encontram? Quem os identificou e quais as fontes históricas que demonstram essa expolição ou aquisição em condições de pressão e de desequilíbrio de poder próprias do período colonial? Enfrentamos, como refere Clémentine Deliss (2020), a incalculabilidade do ato de restituição, ainda mais quando não sabemos sobre o que estamos a tratar.

O desinteresse e a ausência de conhecimento sobre esses bens patrimoniais em Portugal são evidentes no “Inquérito à presença de património proveniente de territórios não europeus nos museus portugueses” que a Comissão Nacional Portuguesa do Conselho Internacional de Museus oportunamente promoveu em 2021. Das mais de quatrocentas instituições museológicas portuguesas, responderam ao seu inquérito 67 museus. Por meio das suas respostas sabemos que uma parte possui coleções não europeias e que muitas destas possuem documentação associada a esses mesmos objetos, o que significa que existem dados que podem conduzir a um conhecimento mais aprofundado sobre a sua proveniência e os contextos de aquisição. O inquérito revelou também a “inexistência de um inventário detalhado”, assim como “um estudo deficitário dessas coleções” numa parte significativa dos museus que aderiram a esse estudo (Amaro, Felismino, 2021, p.133).

Como argumenta Clémentine Deliss – ex-directora do Weltkulturen Museum [Museu das Culturas do Mundo], em Frankfurt, de 2010 a 2015 –, os museus que promoveram a constituição dessas coleções e exposições de artefactos provenientes de contextos culturais sob domínio colonial procuravam moldar as visões dos visitantes relativamente ao progresso ocidental e europeu (Deliss, 2020, p.15-16). As exposições que outrora celebravam esse mesmo “progresso civilizacional ocidental” podem agora servir de prova de violência e da injustiça vivida por aqueles que se encontravam do outro lado desse progresso (Sterling, 2024, p.3). Para isso, é necessário compreender as epistemologias que as produziram e que se refletem, ainda hoje, na forma como as coleções estão organizadas, e muitas vezes ainda no modo como são estudadas e apresentadas ao público. Para essa descodificação dos milhares de artefactos que existem nos museus, incluindo o acesso à informação sobre as práticas e os contextos coloniais de recolha, é preciso contactar com os descendentes de comunidades e as suas diásporas, bem como aceder aos múltiplos depósitos das instituições. Mantidos em unidades de armazenamento muitas vezes inacessíveis, os artefactos adquiridos, roubados, saqueados ou resgatados em nome da ciência, do comércio ou de trocas diplomáticas foram inscritos nos anais históricos das instituições e aí permanecem, invisíveis e silenciados (Deliss, 2020, p.16). Nesses anais é possível recuperar informação histórica fundamental para proceder a estudos de proveniência e reconstituição de itinerários de artefactos culturais passíveis de contribuírem para novas narrativas históricas, discursos museológicos, restituições ou reparações.

Na Europa, muito tem sido publicado nos últimos anos sobre as origens coloniais e os legados dos antigos museus etnológicos e imperiais. Tentativas de descolonizar abrindo os museus à investigação por meio de projetos colaborativos com universidades e comunidades, promovendo a revisão de conteúdos e da linguagem eurocêntrica e racializada replicada pelas instituições museológicas ao longo de décadas, são apenas alguns dos breves exemplos de projetos desenvolvidos. Ao mesmo tempo, novas linhas e centros de investigação surgem no continente africano, nomeadamente em Dakar, Cidade do Cabo ou Nairobi, onde artistas e curadores redefinem com sucesso a museologia e as suas práticas. Vários curadores, artistas e ativistas africanos têm trabalhado em instituições europeias com o desafio de repensar as “coleções etnográficas”, inclusivamente dos seus países de origem. Criam-se lugares de curadoria especializada na temática, como foi o caso do Museu da Universidade de Manchester, onde existe um *curator of living cultures* [curador de culturas vivas].^1^

Em vários países europeus, os debates sobre pesquisa de proveniência suscitaram uma panóplia de iniciativas científicas e museológicas, bem como a formulação de linhas orientadoras e mesmo legislação para coordenar a gestão, a investigação e a restituição de coleções coloniais. Uma mudança geracional e os distanciamentos face ao período colonial estão na origem deste ressurgimento do debate sobre coleções de origens coloniais nos museus do Norte Global (Van Beurden, Gondola, Lacaille, 2023, p.23). Existe, naturalmente, a longa história de pedidos de restituição que consistentemente tem chamado a atenção sobre a origem problemática das coleções coloniais. Essa questão também se conectou com o crescente interesse académico sobre as formas como os passados coloniais sobrevivem hoje em dia e como podemos “confrontar o legado colonial no museu” por meio de várias iniciativas, nomeadamente exposições como a que em 2025 foi inaugurada no Museu Municipal Santos Rocha, em Portugal.^2^ A reinvenção e reinstalação dos museus etnográficos europeus teve igualmente influência nesse ressurgimento. A criação do Humboldt Forum, em Berlim, do Museu do Quai Branly, em Paris, ou a renovação do Africa Museum, em Tervuren, desencadearam inúmeras críticas à forma como as suas exposições mantinham paradigmas coloniais de conhecimento. Em Portugal, o Museu Nacional de Etnologia inaugurou em 2024 a exposição “Desconstruir o colonialismo, descolonizar o imaginário”, com o objetivo de contribuir para uma renovação do conhecimento sobre a questão colonial portuguesa. Contudo, a instituição está ainda a trabalhar para apresentar as suas coleções com uma visão renovada.

Os artigos reunidos neste suplemento, “Coleções coloniais e pós-coloniais em Portugal: reconstituir trajetórias e repensar narrativas”, de *História, Ciências, Saúde - Manguinhos* manifestam a crescente atenção dada, nos últimos anos, à necessidade de revisitar, problematizar e reinterpretar os legados coloniais presentes nas instituições museológicas e científicas em Portugal. Os textos refletem o trabalho que está a ser desenvolvido sobre o legado de várias instituições portuguesas – como o Museu Nacional de História Natural e da Ciência, o Museu Municipal Santos Rocha, o Museu Nacional de Arqueologia e o Museu de História Natural e da Ciência da Universidade do Porto –, evidenciando como as suas coleções foram, em muitos casos, moldadas por dinâmicas de ocupação, missionação, comércio e dominação colonial. Pretendemos igualmente destacar os estudos em curso sobre coleções recolhidas em territórios sob domínio português por meio da atividade missionária, e a forma como o estudo dos itinerários das coleções e das redes de poder associadas à sua constituição permite desvendar as proveniências, os contextos de aquisição e as permanências dos discursos eurocêntricos. Os artigos destacam também a crescente atenção conferida à arte africana contemporânea e aos artistas de ascendência africana cujas obras reconfiguram os modos de representar a história e a memória colonial. A componente do colecionismo privado também é integrada neste suplemento como forma de compreender as motivações, os contextos e os impactos dessas coleções, especialmente quando pertencentes a artistas, e como dialogam com as histórias colonial e pós-colonial portuguesas.

Estes textos resultam da colaboração interinstitucional e da dedicação de investigadores comprometidos com a necessidade de revisão das histórias dominantes, pretendendo apelar à responsabilidade social dos museus e instituições científicas face ao seu património colonial.

## NOTAS

1 O antropólogo Njabulo Chipanguro, natural do Zimbábue, desenvolveu cocuradoria de coleções coloniais entre 2022 e 2025 no Museu de Manchester. Doutorado em Antropologia pela Universidade de Witwatersrand, em Joanesburgo, África do Sul, trabalhou anteriormente como curador no departamento de arqueologia dos Museus e Monumentos Nacionais do Zimbábue durante dez anos. Ver Chipangura, Mataga (2021).

2 “Confrontar o Legado Colonial no Museu” foi o título de uma exposição organizada pelo projeto “TRANSMAT – Materialidades transnacionais (1850-1930): reconstituir coleções e conectar histórias” (PTDC/FER-HFC/2793/2020), em março de 2025, no Museu Municipal Santos Rocha, localizado em Figueira da Foz, Portugal. Durante quatro anos o projeto TRANSMAT estudou as coleções transnacionais do Museu Municipal Santos Rocha e do Museu Nacional de Arqueologia (sobre o projeto, ver Pereira, Carvalho, Cardoso, 2021). O trabalho foi desenvolvido no âmbito do Instituto de História Contemporânea, que é financiado por fundos nacionais através da Fundação para a Ciência e a Tecnologia (FCT), I.P., no âmbito dos projetos UID/04209 e LA/P/0132/2020 (DOI 10.54499/LA/P/0132/2020). Elisabete Pereira recebe financiamento CEEC-FCT (10.54499/2022.04155.CEECIND/CP1734/CT0004), e Quintino Lopes recebe financiamento CEEC-FCT (10.54499/2021.00906.CEECIND/CP1670/CT0005).
